# Medication and Road Test Performance Among Cognitively Healthy Older Adults

**DOI:** 10.1001/jamanetworkopen.2023.35651

**Published:** 2023-09-29

**Authors:** David B. Carr, Kebede Beyene, Jason Doherty, Samantha A. Murphy, Ann M. Johnson, Hailee Domash, Noah Riley, Alexis Walker, Ashwin Sabapathy, John C. Morris, Ganesh M. Babulal

**Affiliations:** 1 Department of Medicine, Washington University in St Louis, St Louis, Missouri; 2 Department of Pharmaceutical and Administrative Sciences, University of Health Sciences and Pharmacy in St Louis, St Louis, Missouri; 3 Department of Neurology, Washington University in Saint Louis School of Medicine, St Louis, Missouri; 4 Center for Clinical Studies, Washington University School of Medicine, St Louis, Missouri; 5 Institute of Public Health, Washington University School of Medicine, St Louis, Missouri; 6 Department of Psychology, Faculty of Humanities, University of Johannesburg, Johannesburg, South Africa; 7 Department of Clinical Research and Leadership, The George Washington University School of Medicine and Health Sciences, Washington, DC

## Abstract

**Importance:**

Older adults are increasingly prescribed medications that have adverse effects. Prior studies have found a higher risk of motor vehicle crashes to be associated with certain medication use.

**Objective:**

To determine whether specific medication classes were associated with performance decline as assessed by a standardized road test in a community sample of cognitively healthy older adults, to evaluate additional associations of poor road test performance with comorbid medical conditions and demographic characteristics, and to test the hypothesis that specific medication classes (ie, antidepressants, benzodiazepines, sedatives or hypnotics, anticholinergics, antihistamines, and nonsteroidal anti-inflammatory drugs or acetaminophen) would be associated with an increase in risk of impaired driving performance over time.

**Design, Setting, and Participants:**

This was a prospective cohort study of 198 cognitively healthy adults 65 years and older with a valid driver’s license who were followed up annually, with rolling enrollment. Data were collected from participants in St Louis, Missouri, and neighboring Illinois who were enrolled in the Knight Alzheimer’s Disease Research Center. Data were collected from August 28, 2012, to March 14, 2023, and analyzed from April 1 to 25, 2023. Participants with healthy cognition, defined as a Clinical Dementia Rating score of 0 at baseline and subsequent visits, who had available clinical, neuropsychological, road tests, and self-reported medication data were included.

**Exposure:**

Potentially driver-impairing medication use.

**Main Outcomes and Measures:**

The primary outcome measure was performance on the Washington University Road Test (pass or marginal/fail). Multivariable Cox proportional hazards models were used to evaluate associations between potentially driver-impairing medication use and road test performance.

**Results:**

Of the 198 included adults (mean [SD] baseline age, 72.6 [4.6] years; 87 female [43.9%]), 70 (35%) received a marginal/fail rating on the road test over a mean (SD) follow-up of 5.70 (2.45) years. Any use of antidepressants (adjusted hazard ratio [aHR], 2.68; 95% CI, 1.69-4.71), serotonin and norepinephrine reuptake inhibitors (aHR, 2.68; 95% CI, 1.54-4.64), sedatives or hypnotics (aHR, 2.70; 95% CI, 1.40-5.19), or nonsteroidal anti-inflammatory drugs (aHR, 2.72; 95% CI, 1.31-5.63) was associated with an increase in risk of receiving a marginal/fail rating on the road test compared with control individuals. Conversely, participants taking lipid-lowering agents had a lower risk of receiving a marginal/fail rating compared to control individuals. There were no statistically significant associations found between anticholinergic or antihistamines and poor performance.

**Conclusions and Relevance:**

In this prospective cohort study, specific medication classes were associated with an increase in risk of poor road test performance over time. Clinicians should consider this information and counsel patients accordingly when prescribing these medications.

## Key Points

**Question:**

What potentially driver-impairing medications are associated with poor road test performance over time?

**Findings:**

In this cohort study of 198 cognitively healthy drivers 65 years and older, antidepressants (including selective serotonin and norepinephrine reuptake inhibitors), sedatives or hypnotics, and nonsteroidal anti-inflammatory drugs or acetaminophen medication categories were associated with a higher risk of failing a road test over time. There were no statistically significant associations found between anticholinergic or antihistamines and poor performance.

**Meaning:**

The findings in this study suggest that physicians and pharmacists should be aware of potential driving risks in older drivers who are prescribed psychotropic drugs and pain medications and provide consultation accordingly.

## Teaser

This cohort study evaluates associations between different potentially driver-impairing medication classes and road test results among adults 65 years and older.

## Introduction

Nearly 17% of the US population is aged 65 years and older.^1^ Of these 50 million individuals, more than 48 million are licensed drivers.^2^ By 2050, 25% of all licensed drivers are estimated to be 70 years and older.^3^ Drivers 70 years and older are retaining their driver licenses longer and driving more miles than ever.^2^ A driver 65 years or older has an average driving life expectancy of 10 or more years.^4^ The ability to drive independently supports community mobility, social connectedness, and critical access to health care.

Adults 65 years and older (hereafter, older adults) have an increased risk of automotive crash compared with middle-aged drivers when accounting for exposure or miles driven annually.^5^ Decline in driving performance is associated with older age,^6^ where motor vehicle crashes remain a leading cause of injury and death in older adults.^7^ In 2020, a daily average of 20 older adults were killed and 540 injured in motor vehicle crashes nationwide.^8^ Driving fatalities increase with age, and this elevated susceptibility has been attributed to increasing frailty,^9^ although the type of crash, number and type of vehicles involved, and road conditions contribute additional risk. Cognitive disorders remain a major risk factor.^10^

The US Department of Transportation and National Highway Traffic Safety Administration^11^ reported that more than 90 medication classes were associated with motor vehicle crashes among older drivers. Medications commonly associated with driving impairment cause sedation, drowsiness, reduced motor coordination, hypoglycemia, blurred vision, hypotension, syncope, and ataxia.^12^ Notable among these are antidepressants, benzodiazepines, sedatives and hypnotics, antihistamines, opioids, antipsychotics, and anticholinergics. Our prior publication^13^ contains detailed information on medications linked with driving impairment and motor vehicle crashes. The extant literature has categorized these classes as potentially driver-impairing medications that are more prevalently taken by older adults.^14^ However, based on the limited evidence and conventional methodology, it is difficult to determine whether an elevated crash risk is due to medication adverse effects, the medical condition being treated, or other medications or comorbid conditions.

Among systematic meta-analyses^15,16,17,18^ in older adult samples, poorer driving performance was consistently identified for specific medical conditions (eg, dementia, Parkinson disease, and stroke). Drivers 65 years and older who are racialized as Black have a higher risk of driving reduction, mobility restriction, and driving cessation compared to their non-Hispanic White counterparts.^19,20^ Given the projected growth of the aging population, number of crashes and injuries among older drivers, and risk of polypharmacy and multimorbidity, driving ability and safety remain top public health priorities. The primary aim of this study was to determine whether certain classes of medications (antipsychotics, antidepressants, benzodiazepines, sedatives and hypnotics, opioids, anticholinergics, antihistamines, nonsteroidal anti-inflammatory drugs [NSAIDs]/acetaminophen) were associated with driving impairment as assessed by a performance-based road test in a sample of cognitively healthy older adults. As a secondary aim, we investigated the association between additional classes of medications (anticoagulants/antiplatelets, anticonvulsants, antidiabetic, lipid-lowering agents, and sympathomimetic and sympatholytic agents) and driving performance.

## Methods

### Study Design and Sample

Community-dwelling adults 65 years and older were enrolled in longitudinal studies of aging, dementia, and driving at the Knight Alzheimer Disease Research Center^21,22^ and the Driving Real-world In-Vehicle Evaluation System (DRIVES) project.^23^ In this sample, participants were drawn from parent studies if they had completed at least 2 standardized road tests, were cognitively healthy at baseline defined as a score of 0 on the Clinical Dementia Rating scale,^24^ had a valid driving license, resided in the St Louis, Missouri, metropolitan area or neighboring Illinois, and drove at least once a week. During annual visits (from baseline), data were collected, including clinical, neurological, neuropsychological, and functional assessments with high (>90% retention rates). Among the included participants, data were collected from August 28, 2012, to March 14, 2023, and analyses were conducted from April 1 to 25, 2023. Ethics approval was obtained from the Human Research Protection Office of Washington University in St Louis, St Louis, Missouri, and written informed consent was obtained from all participants. This prospective cohort study followed the Strengthening the Reporting of Observational Studies in Epidemiology (STROBE) reporting guideline.

### Road Test

The Washington University road test^25^ consists of a 12-mile public, in-traffic route designed to encapsulate a wide range of traffic conditions, road types, and intersectional navigation scenarios. The test is conducted in a 4-door midsize sedan fitted with dual brakes and mirrors if intervention is required to prevent a crash. Driving performance was assessed annually by a professional driving instructor seated in the passenger-side front seat. The examiner provides a rating of pass, marginal, or fail. A pass rating demonstrates competency in driving behavior with minimal errors and no safety concerns. A marginal rating indicates errors with low to moderate risk for safety concerns (eg, inconsistent speed or rolling stops), while a fail rating indicates moderate to large risk (eg, drifting out of lane, running stop light/sign, or excessive speeding). Failure on the road test by cognitively healthy older adults is rare, as demonstrated by prior studies and, as a result, both fail and marginal outcomes are typically combined into a single category (marginal/fail) to analyze safe (no risk) vs unsafe (any risk) driving.^26,27,28,29^

### Comorbidity Index

Disease comorbidity was assessed using the Charlson Comorbidity Index (CCI).^30^ Updated weightings from Quan et al were used.^31^

### Potentially Driver-Impairing Drug Categories

Medication data were collected at annual office visits per the National Alzheimer Coordinating Center Uniform Data Set guidelines.^32^ A clinician interviewed participants and obtained self-reported medications taken within the past 2 weeks. Use was defined by a participant endorsing ever taking a potentially driver-impairing medication during subsequent follow-up. We used the American Hospital Formulary Service^33^ classification system to group all prescribed and over-the-counter medications into classes that reflect similar pharmacologic and therapeutic characteristics. Classification was completed by a pharmacist (K.B.) with significant clinical and data-linking experience. All drug classes were reviewed to identify the putative potentially driver-impairing medication categories as defined by the US Food and Drug Administration^34^ and previous studies.^13,14,35^ A comprehensive list of all medications in this study, their respective classification system, and potentially driver-impairing categories are available in eTables 1 and 2 in Supplement 1.

### Cognition and Vision

Objective cognitive status was assessed using 4 measures that were then combined into a composite score. The subtests consisted of Trailmaking A^36^ to assess processing speed, Trailmaking B^36^ to assess executive function, Free and Cued Selective Reminding Task: Free Recall Score^37^ to assess episodic memory, and animal fluency^38^ to assess semantic memory. The preclinical Alzheimer cognitive composite (PACC)^39^ score was calculated by standardizing the scores of the subtasks using their means and SDs and then calculating the mean of each participant’s standardized score.

Vision was assessed using the app-based King-Devick visual acuity test. Participants read strings of letters in decreasing text size presented on the tablet. The tablet was positioned approximately 40 cm from the participant’s face for near acuity and 2 meters for far acuity. The total number of letters correctly read in near and far tasks was recorded as their visual acuity score for each distance. Far acuity is more crucial for observing vehicles, pedestrians, traffic lights, signage, and traffic flow to ensure safe driving.^40^

### Neighborhood Deprivation

Socioeconomic economic status was assessed by assigning an area of deprivation index (ADI)^41^ score based on participants’ home address at baseline. The ADI ranks neighborhoods by socioeconomic disadvantage and is based on 17 factors, including income, employment, and quality of housing, and is categorized on a census-block level. Participants’ home addresses were geocoded using an application programming interface provided by geocodio.io and merged with ADI rankings. Since our sample included participants residing across 2 states, national-level rankings were used in analyses.

### Statistical Analysis

Baseline sample characteristics were compared using χ^2^ test and independent samples *t* test. Continuous variables are described as means with SDs and categorical variables as counts and percentages. The incidence of receiving a marginal/fail rating on the annual road test was determined based on the number of marginal/failures per 100 person-years of follow-up while taking a potentially driver-impairing medication. Since participants enrolled in the study at different times and were followed up for differing periods, Kaplan-Meier curves were used to estimate the cumulative probability of receiving a marginal/failing rating during the follow-up period, stratified by potentially driver-impairing medication use.

Differences in cumulative probabilities were assessed using log-rank tests. Separate multivariable Cox proportional hazards models examined the independent association between potentially driver-impairing medication categories (antidepressants, serotonin and norepinephrine reuptake inhibitors [SNRIs] and selective serotonin reuptake inhibitors [SSRIs], sedative and hypnotic agents, NSAIDs or acetaminophen, or anticholinergics or antihistamines) on annual road test performance. A secondary set of analyses was conducted on 6 additional and uncommon potentially driver-impairing medications. Medications prescribed for a small number of participants (eg, skeletal muscle relaxants, antipsychotics, opioids, central nervous system stimulants, and cholinergic agents) were not selected for analyses. Preliminary analyses confirmed these medication classes contained a small number of observations, which is not optimal to study predictors of the target outcome. All Cox models were adjusted for sex, age, race, CCI, ADI, PACC, and far visual acuity (prioritized over near), all of which have been observed to be associated with driving outcomes.^4,19,42^ Race was self-reported according to the 5 minimum categories from the Office of Management and Budget (American Indian or Alaska Native, Asian, Black or African American, Native Hawaiian or Other Pacific Islander, and White). Due to a strong degree of collinearity, we did not include near and far visual acuity scores in the same model. A separate model substituted far with near visual acuity. The proportional hazards assumption was assessed using Schoenfeld residuals; all variables in the Cox proportional hazards models met this assumption. Results are presented as adjusted hazard ratios (aHRs) and 95% CIs. *P* values <.05 were considered statistically significant. As a sensitivity analysis, we conducted competing risk analyses for medications included in primary and secondary analyses using Fine-Gray subdistribution hazard ratio models.^43^ Complete case analysis was carried out as missing data were minimal. Data processing and merging were completed using R version 4.2.0 (R Foundation), and STATA version 14.0 (StataCorp) was used for statistical analyses and figure generation.

## Results

### Participant Demographic Characteristics

A total of 198 cognitively healthy older individuals were included in the study, contributing to 861 person-years of follow-up time (Table 1). The mean (SD) follow-up was 5.70 (2.45) years, while the range varied from 1.00 to 10.24 years. The mean (SD) age was 72.6 (4.6) years, and 87 participants (43.9%) were female. Nineteen participants (9.6%) identified as Black and 178 (90.4%) identified as White. No other groups were represented. Most participants (88.4%) had at least some college education. Participants’ mean (SD) PACC score was 0 (0.7), and the mean (SD) far visual acuity score was 51.1 (5.2). The mean (range) ADI national ranking was 42.5 (3-99; lower indicates more deprivation), while 181 participants (91.4%) had CCI scores of 0, indicating no serious comorbidities. When compared to participants who passed the road test, those who received a marginal/fail rating on the road test had lower near visual acuity scores (mean [SD], 66.2 [4.3] vs 63.8 [5.6]; *P* = .001) at baseline. In contrast, those who received marginal/fail rating on a road test had lower CCI scores than those who passed the road test. No statistically significant differences were observed for other baseline characteristics. There were 8 participants who progressed to Clinical Dementia Rating score 0.5 or greater over the course of the study; however, there were no statistically significant differences in their road test performance or medication class compared to those who maintained a Clinical Dementia Rating score of 0.

**Table 1.** Participant Baseline Characteristics by Road Test Performance

Characteristic	No. (%)			*P* value
	Total (N = 198)	Marginal/fail rating, no (n = 128 [64.6%])	Marginal/fail rating, yes (n = 70 [35.4%])	
Sex				
Female	87 (43.9)	53 (41.4)	34 (48.6)	.33^a^
Male	111 (56.1)	75 (58.6)	36 (51.4)	
Age, y				
65-69	71 (35.9)	50 (39.1)	21 (30)	.09^a^
70-74	72 (36.4)	49 (38.3)	23 (32.9)	
>75	55 (27.8)	29 (22.7)	26 (37.1)	
Race^b^				
Black	19 (9.6)	12 (9.4)	7 (10)	.90^a^
White	178 (90.4)	115 (90.6)	63 (90)	
Education				
High school graduate/GED	23 (11.6)	15 (11.7)	8 (11.4)	.87^a^
College and above	175 (88.4)	113 (88.3)	62 (88.6)	
CCI				
0	181 (91.4)	113 (88.3)	68 (97.1)	.03^a^
≥1	17 (8.6)	15 (11.7)	2 (2.9)	
PACC score, mean (SD)	0.0 (0.7)	0.0 (0.6)	−0.1 (0.8)	.39^c^
Visual acuity (near), mean (SD)	65.3 (4.9)	66.2 (4.3)	63.8 (5.6)	.001^c^
Visual acuity (far), mean (SD)	51.1 (5.2)	51.6 (5.2)	50.4 (5.2)	.14^c^
ADI, mean (SD)	42.5 (23.2)	42.3 (23.1)	42.7 (23.6)	.92^c^
Age, mean (SD), y	72.6 (4.6)	72.1 (4.6)	73.3 (4.7)	.08^c^

Abbreviations: ADI, area deprivation index national rank; CCI, Charlson comorbidity index; GED, General Educational Development; PACC, Preclinical Alzheimer Cognitive Composite score.

^a^ χ^2^.

^b^ Data on race were self-reported and collected to evaluate disparities by racialization. Participants in this study identified as Black or White only; no other groups were identified.

^c^ *t* test.

### Potentially Driver-Impairing Drugs and Kaplan Meier Assessment

**Figure 1.**
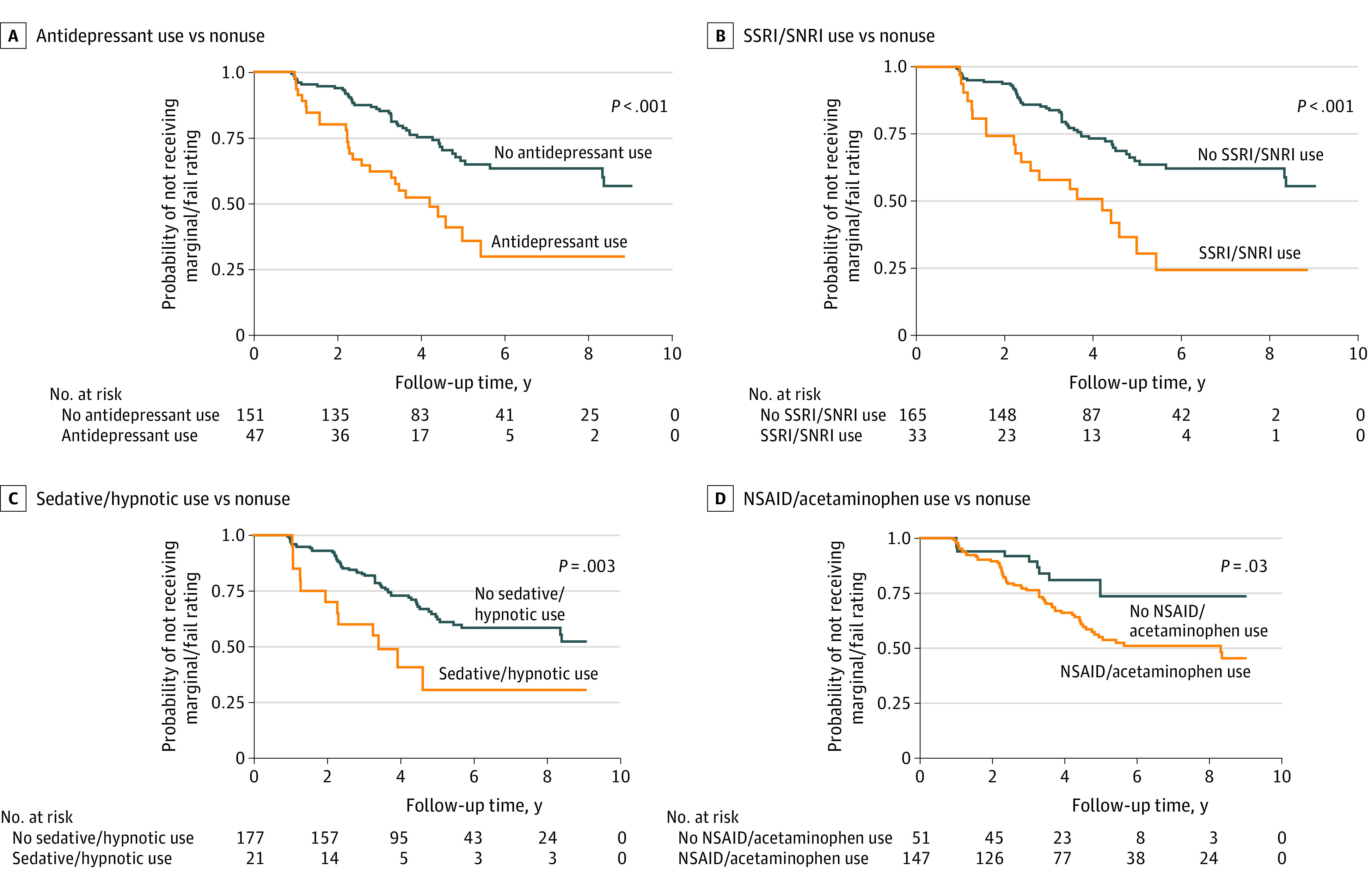
Four Potentially Impairing Drug Categories and Road Test Outcome Probabilities NSAID indicates nonsteroidal anti-inflammatory drugs; SNRI, serotonin and norepinephrine reuptake inhibitors; SSRI, selective serotonin reuptake inhibitors.

**Figure 2.**
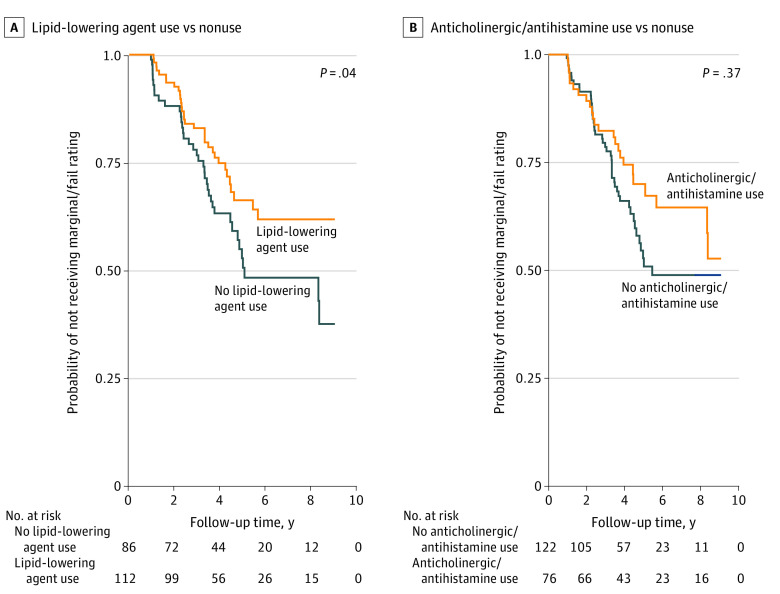
Lipid-Lowering Drug and Anticholinergic/Antihistamines Classes and Road Test Outcome Probabilities

Prescribed and over-the-counter medications during the study period are summarized in Table 2. Drugs impacting the central nervous system were the most frequently prescribed potentially driver-impairing categories (164 respondents [82.8%] endorsed central nervous system drug use), followed by cardiovascular or hypertension drugs (160 [80.8%]) and gastrointestinal drugs (95 [48.0%]). A total of 70 participants received a marginal/fail rating on a road test during the 10-year follow-up period, and the incidence rate of marginal/fail was 8.1-per 100 person-years for the cohort (Table 3). Kaplan Meier analysis and log-rank tests indicated that antidepressants, SSRIs or SNRIs, sedatives or hypnotics, and NSAID or acetaminophen use was associated with a higher risk of receiving a marginal/fail rating on a road test compared with nonuse (Figure 1). Conversely, lipid-lowering agents were associated with a lower risk of receiving a marginal/fail rating compared to nonuse (Figure 2).

**Table 2.** Most Common Potentially Driver-Impairing Medications Prescribed to Study Participants During the Study Period^a^

Drug class^b^	No. (%)		
	Total (N = 198)	Marginal/fail rating, no (n = 128)	Marginal/fail rating, yes (n = 70)
**Medications included in primary analysis**			
Any antidepressant	21 (16.4)	26 (37.1)	47 (23.7)
SSRIs/SNRIs	13 (10.2)	20 (28.6)	33 (16.7)
Any sedative or hypnotic agent	9 (7.0)	12 (17.1)	21 (10.6)
NSAIDs/acetaminophen	86 (67.2)	61 (87.1)	147 (74.2)
Anticholinergics/antihistamines	56 (43.8)	28 (40)	84 (42.4)
**Medications included in secondary analysis**			
Anticoagulants/antiplatelets	18 (14.1)	8 (11.4)	26 (13.1)
Anticonvulsants	14 (10.9)	12 (17.1)	26 (13.1)
Antidiabetic medications	13 (10.2)	8 (11.4)	21 (10.6)
Lipid-lowering agents	80 (62.5)	32 (45.7)	112 (56.6)
Sympathomimetic agents	23 (18)	13 (18.6)	36 (18.2)
Sympatholytic agents	12 (9.4)	12 (17.1)	24 (12.1)

Abbreviations: NSAIDs, nonsteroidal anti-inflammatory drugs; SNRIs, serotonin and norepinephrine reuptake inhibitors; SSRIs, selective serotonin reuptake inhibitors.

^a^ Drugs are grouped based on the American Hospital Formulary Service pharmacologic-therapeutic classification system.

^b^ Participants could report more than one medicine, so percentages exceed 100%.

**Table 3.** Incidence Rates and Adjusted Hazard Ratios for Risk of Marginal/Fail Rating on Road Test by Potentially Driver-Impairing Drug Categories

Drug^a^	No. in each group	Time at risk, y	No. of marginal/fail rating^b^	No. of marginal/fail rating per 100 person-years	HR (95% CI)^c^^,^^d^	*P* value
Any antidepressant						
No	151	695.7	44	6.3	2.82 (1.69-4.71)	<.001
Yes	47	164.7	26	15.8		
SSRI/SNRI						
No	165	746.6	50	6.7	2.68 (1.54-4.64)	<.001
Yes	33	113.8	20	17.6		
Sedative and hypnotic agents						
No	177	788.6	58	7.4	2.72 (1.41-5.22)	.003
Yes	21	71.8	12	16.7		
NSAIDs/acetaminophen						
No	51	205.4	9	4.4	2.72 (1.31-5.63)	.007
Yes	147	655.0	61	9.3		
Anticholinergic/antihistamines						
No	114	469.3	42	8.9	0.84 (0.51-1.38)	.49
Yes	84	391.1	28	7.2		
Total	198	860.4	70	8.1	NA	NA

Abbreviations: HR, hazard ratio; NA, not applicable; NSAIDs, nonsteroidal anti-inflammatory drugs; SNRIs, serotonin and norepinephrine reuptake inhibitors; SSRIs, selective serotonin reuptake inhibitors.

^a^ For all drugs, the no response option served as the reference group for Cox proportional hazards regression analyses.

^b^ Number of marginal/fail rating refers to the number of participants who received marginal/fail rating on a road test.

^c^ HRs were adjusted for sex, age, education, area of deprivation index, Charlson comorbidity index, Preclinical Alzheimer Cognitive Composite score, and visual acuity (far) score.

^d^ For Cox regression, marginal/fail rating on road test (no = 0) served as the reference group.

### Multivariable Survival Analyses

After adjusting for sex, age, race, education, ADI, CCI, PACC, and far visual acuity, any antidepressant use was associated with a 2.8-fold increased risk of a marginal/fail rating (aHR = 2.82; 95% CI, 1.69-4.71). When modeling exposure according to SSRI/SNRI use alone, the use of SSRI/SNRI medications was significantly associated with the risk of a marginal/fail rating (aHR = 2.68; 95% CI, 1.54-4.64). Similarly, the risk of a marginal/fail rating on a road test was higher among those taking sedative and hypnotic agents compared with nonuse (aHR = 2.72; 95% CI, 1.41-5.22). The use of any NSAIDs/acetaminophen (aHR = 2.72; 95%CI, 1.31-5.63) was significantly associated with an increase in risk of a marginal/fail rating on the road test. We did not find a significant effect between anticholinergics/antihistamines and driving performance (Table 3). Supplanting near visual acuity did not significantly change any results (eTable 4 in Supplement 1).

In the secondary analysis of uncommon potentially driver-impairing drugs, sympatholytic agents, anticonvulsants, and antidiabetics were associated with an increase in risk of a marginal/fail rating on the road tests but this increase was not statistically significant. Conversely, sympathomimetic agents, anticoagulants or antiplatelets, and lipid-lowering agents were negatively associated with risk of receiving a marginal/fail rating on the road tests but this was not statistically significant either (eTable 3 in Supplement 1). The competing risk analysis results remained consistent with the standard Cox regression results (eTable 5 in Supplement 1).

### Discussion

This cohort study found that in a cognitively healthy, community-residing sample of adults 65 years and older who were taking SSRI or SNRIs, antidepressants, sedatives or hypnotics, or NSAIDs or acetaminophen were found to have a higher risk of driving impairment on a road test (marginal/fail rating) compared to nonuse. These hazard ratios were robust after adjusting for age, sex, race, education, neighborhood deprivation, comorbidities, cognitive functioning, and visual acuity score. These results suggest that the potentially driver-impairing medication classes may increase the risk for poor driving performance over time among older drivers.

These findings are consistent with previous studies published in the literature that found associations with motor vehicle crashes and antidepressants^44,45^ and sedatives and hypnotics.^46,47^ Other medications that have been implicated in the literature include anticonvulsants, muscle relaxants, and anticholinergic drugs. These were either not found to be associated with driving impairment in our study or the prevalence of the drug was too small to study (eg, opioids or muscle relaxants). It is possible that the number of older adults taking anticholinergic medications in our sample was too small to observe an effect, the actual medications taken in that specific class were less likely to be associated with impairment (eg, nonsedating antihistamine), or perhaps a more sensitive outcome measure like a driving simulator or naturalistic driving would have picked up on more subtle impairments.

The negative association between psychoactive drug use and driving performance may be explained by the effect of these drugs on neurotransmitters, such as noradrenaline, serotonin, histamine, acetylcholine, and GABA.^48^ Tricyclic antidepressants work by inhibiting the reabsorption of serotonin, dopamine, and norepinephrine but also block postsynaptic α_1_-adrenergic, histaminergic, and muscarinic receptors. This nonselective action may lead to a number of adverse effects, including dizziness, drowsiness, attention deficit, cognitive difficulties, and psychomotor impairment.^49^ SSRIs exert their pharmacological action by selectively inhibiting the reuptake of serotonin, increasing the level of this neurotransmitter in the synapse.^49^ Due to their selective inhibition, SSRIs have milder adverse effects than older antidepressants. However, some common side effects of SSRI use (eg, sleep disturbance, agitation, dizziness, headache, and fatigue) lead to driving impairment. Antidepressants (eg, SSRIs and SNRIs) can also inhibit cytochrome P450 (CYP450), a liver enzyme responsible for the metabolism of several drugs, including a number of other potentially driver-impairing medications.^50^ This drug-drug interaction can potentially increase the risk of adverse effects of other potentially driver-impairing medications and hence hazardous driving. Sedative and hypnotic agents are commonly used to treat sleep disorders and anxiety in older adults. Benzodiazepines are one of the most frequently prescribed medications within this group. While benzodiazepines bind to specific sites on the GABA_A_ receptor in the brain, which enhances the activity of GABA, a neurotransmitter that inhibits brain activity, they bind nonselectively.^51^ As a result, benzodiazepines (including hypnotics) use may cause sedation, impaired motor coordination, and drowsiness, and this can impair drivers’ ability to focus and react quickly to changes in the environment, such as other vehicles, pedestrians, or traffic signals. Benzodiazepine can also cause blurred vision due to inhibition of the GABAergic system in the retina and visual cortex, which can further affect driving ability. Most NSAIDs have little impact on driving if they are taken correctly. However, NSAIDs have several adverse effects, including dizziness or lightheadedness, drowsiness, vision impairments, and difficulty concentrating, that can affect driving ability.^52^ This may explain the negative association observed between NSAID use and performance on the road test. Long-term use of NSAIDs by older patients is common and could result in drug-drug interactions that exacerbate existing medical conditions known to contribute to driving impairment, such as hypertension and heart failure.^53,54^

Few studies have reported on the potential benefits of medications on driving, which is surprising since medications for Parkinson disease, epilepsy, and pain have the potential to improve driving. One study^55^ found that drivers taking statins had a decreased motor vehicle crash risk compared to those who were nonadherent; adherent patients were more likely to engage in healthy behaviors. However, numerous studies have suggested the protective effects of statin on cognitive functioning,^56^ which could have a positive impact on driving.

### Limitations

This study has limitations. The predominately non-Hispanic White sample with predominantly high education limits generalizability. No data were collected on medication adherence, dosage, frequency, or route. Longitudinal changes in potentially driver-impairing medications were not assessed since medications may have been prescribed, taken, and then deprescribed within a year, which would be missed during the annual visit based on the 2-week time frame. Road tests are limited based on the observations of the examiner and may not detect more subtle behaviors that can be demonstrated in crash scenarios via driving simulator or by documenting motor vehicle crash data in a larger sample. Driving reduction and cessation were not assessed in this study since participants were required to be active drivers. Participants with poor driving performance may have preclinical Alzheimer disease, but this was not confirmed with biomarker data. CCI was not associated with poor driving performance in Cox models. It is possible a more robust index that considers disease severity (eg, multimorbidity weighted index)^57,58^ would have had a stronger association. The short-term (hours) and long-term (months) effects of potentially driver-impairing medication on the road tests were not collected to be included in the models. Due to a relatively low number of participants using certain medications known to affect driving performance, such as opioids (n = 14) and antipsychotics (n = 3) we could not assess their association with driving performance. Moreover, it is difficult to separate the effect of potentially driver-impairing medication on driving from the potential effects of the treated diseases: it is possible that the increased risk from potentially driver-impairing medication is lower than the risk of the underlying disease.

## Conclusions

In this prospective cohort study of older adult drivers, antidepressants, SSRIs or SNRIs, sedatives or hypnotics, and NSAIDs or acetaminophens were associated with an increased risk of poor driving performance on a road test. While we cannot determine whether these medications directly caused the risk of decline in driving performance individually or collectively, our results raise concern about the potential negative impact of potentially driver-impairing medications on driving performance. Clinicians and pharmacists should be aware that patients who are prescribed these drugs could be at an increased risk of driving impairment. A clear discussion and review of medications in relation to the driving task should be included in the care of older adults. It is understood that potentially driver-impairing medications may not be avoidable in some cases, given limited alternatives. Clinicians might consider following prescribing guidelines such as the Beers criteria,^59^ which could limit adverse effects in older adults and potentially benefit traffic safety. Researchers should consider more studies that examine the potential harm or benefits of medications on the driving. This could be interrogated via in vivo naturalistic driving methodologies and accounting for medication-taking behaviors, substance and alcohol use, and psychological and behavioral factors.

## Notes

Supplement 1. **eTable 1.** Medications included in primary analysis **eTable 2.** Medications included in secondary analysis **eTable 3.** Incidence rates and adjusted hazard ratios for risk of marginal/fail rating on road test (i.e. event) by PDI drug categories (N=198) **eTable 4.** Incidence rates and adjusted hazard ratios for risk of marginal/fail rating on road test (i.e. event) by PDI drug categories (N=198) **eTable 5.** Adjusted hazard ratios for marginal/fail rating on a road test estimated with the Fine–Gray subdistribution hazard analysis (N=198)

Supplement 2.
Data Sharing Statement
