# Error in Table

**DOI:** 10.1001/jamanetworkopen.2023.42461

**Published:** 2023-10-25

The Original Investigation titled “Medication and Road Test Performance Among Cognitively Healthy Older Adults,”^1^ published on September 29, 2023, was corrected to fix the headers in Table 2, which had been misordered.
