# Supplementary material for: Medication and Road Test Performance Among Cognitively Healthy Older Adults
Source: JAMA Netw Open. 2023 Sep 29;6(9):e2335651. doi: 10.1001/jamanetworkopen.2023.35651 (PMC10543136; doi:10.1001/jamanetworkopen.2023.35651)
Supplement: Supplement 1. — eTable 1. Medications included in primary analysis eTable 2. Medications included in secondary analysis eTable 3. Incidence rates and adjusted hazard ratios for risk of marginal/fail rating on road test (i.e. event) by PDI drug categories (N=198) eTable 4. Incidence rates and adjusted hazard ratios for risk of marginal/fail rating on road test (i.e. event) by PDI drug categories (N=198) eTable 5. Adjusted hazard ratios for marginal/fail rating on a road test estimated with the Fine–Gray subdistribution hazard analysis (N=198) [file jamanetwopen-e2335651-s001.pdf]

Supplemental Online Content

Carr DB, Beyene K, Doherty J, et al. Medications and road test performance among cognitively unimpaired older adults. *JAMA Netw Open*. 2023;6(9):e2335651. doi:10.1001/jamanetworkopen.2023.35651

eTable1. Medications included in primary analysis

eTable2. Medications included in secondary analysis

eTable 3. Incidence rates and adjusted hazard ratios for risk of marginal/fail rating on road test (i.e. event) by PDI drug categories (N=198)

eTable 4. Incidence rates and adjusted hazard ratios for risk of marginal/fail rating on road test (i.e. event) by PDI drug categories (N=198)

eTable 5. Adjusted hazard ratios for marginal/fail rating on a road test estimated with the Fine–Gray subdistribution hazard analysis (N=198)

This supplemental material has been provided by the authors to give readers additional information about their work.

© 2023 Carr DB et al. *JAMA Network Open.*

**eTable1.** Medications included in primary analysis*	
	**Class (if available)**<br>**Medication**
Antidepressants	amitriptyline, bupropion, citalopram desvenlafaxine, doxepin, duloxetine, escitalopram, fluoxetine, imipramine, mirtazapine, nefazodone, nortriptyline, paroxetine, phenelzine, sertraline, trazodone, venlafaxine, vilazodone
Selective serotonin reuptake inhibitor/Serotonin–norepinephrine reuptake inhibitor	citalopram, desvenlafaxine, duloxetine, escitalopram, fluoxetine, paroxetine, sertraline, venlafaxine, vilazodone
Sedatives and hypnotic agents	<u>Barbiturates</u><br>atropine/hyoscyamine/phenobarbital/scopolamine, butalbital, butalbital/acetaminophen/caffeine, secobarbital
	<u>Benzodiazepines</u><br>alprazolam, chlordiazepoxide, diazepam, lorazepam, temazepam, triazolam<br><u>Non-Benzo Hypnotics/Sedatives</u><br>Buspirone, eszopiclone, suvorexant, zaleplon, zolpidem
NSAIDs/Acetaminophen	acetaminophen, acetaminophen/caffeine, acetaminophen/codeine acetaminophen/diphenhydramine, acetaminophen/hydrocodone acetaminophen/phenyltoloxamine, acetaminophen/propoxyphene acetaminophen/pseudoephedrine, acetylsalicylic acid/sodium bicarbonate/anhydrous citric acid, acetaminophen/ acetylsalicylic acid /caffeine, acetaminophen/butalbital/caffeine, acetaminophen/caffeine acetylsalicylic acid, acetylsalicylic acid/caffeine/ propoxyphene acetylsalicylic acid/dipyridamole, celecoxib, diclofenac, etodolac, ibuprofen, indomethacin, ketorolac, meloxicam, nabumetone, naproxen, piroxicam, rofecoxib
Anticholinergics/Antihistamines	<u>Antihistamines</u><br>acetaminophen/diphenhydramine, acetaminophen/phenyltoloxamine, allergy shots, atropine/chlorpheniramine/hyoscyamine/pseudoephedrine/scopolamine, azelastine, carbinoxamine/methscopolamine/pseudoephedrine, cetirizine, chlorpheniramine/phenylephrine, chlorpheniramine/acetaminophen/phenylephrine, cyproheptadine, dexbrompheniramine/pseudoephedrine, desloratadine, diphenhydramine<br>doxylamine, fexofenadine, ketotifen ophthalmic solution, levocetirizine, loratadine, olopatadine
	<u>H2 Antagonists</u><br>cimetidine, famotidine, ranitidine
	<u>Skeletal Muscle Relaxants</u><br>baclofen, carisoprodol, cyclobenzaprine, methocarbamol, orphenadrine, tizanidine
	<u>Antispasmodics/Antimuscarinics</u>

	atropine, atropine/diphenoxylate, atropine/chlorpheniramine/hyoscyamine/pseudoephedrine/scopolamine, atropine/hyoscyamine/phenobarbital/scopolamine, carbinoxamine/methscopolamine/pseudoephedrine, chlordiazepoxide/clidinium, dicyclomine, diphenoxylate/atropine, fluticasone/umeclidinium/vilanterol, hyoscyamine, ipratropium bromide ipratropium nasal spray, scopolamine, tiotropium, tiotropium/olodaterol, umeclidinium/vilanterol
	<u>Antidiarrheal agents</u><br>atropine/diphenoxylate, bismuth subsalicylate, loperamide
	<u>Smooth muscle relaxants</u><br>Darifenacin, mirabegron, oxybutynin, solifenacin, tolterodine, trospium
Notes: All brand names are converted to generic names	

*This table only includes PDI medications endorsed by the study participants. We did not include all medicines that could potentially affect driving performance.

© 2023 Carr DB et al. *JAMA Network Open.*

**eTable2. Medications included in secondary analysis***	
**Categories**	**<u>Class</u> (if available)**<br>**Medication**
Lipid-lowering agents	alirocumab, atorvastatin, atorvastatin/amlodipine, cholestyramine, colesevelam, docosahexaenoic acid, ezetimibe, ezetimibe/simvastatin, fenofibrate, gemfibrozil, lovastatin<br>lovastatin/niacin, niacin, pitavastatin, pravastatin, rosuvastatin, simvastatin
Antidiabetics	alogliptin, canagliflozin, dapagliflozin, dulaglutide, empagliflozin, exenatide, glimepiride glipizide, glyburide, insulin aspart, insulin degludec, insulin detemir, insulin glargine, insulin lispro, linagliptin, metformin, metformin/sitagliptin, pioglitazone, saxagliptin, sitagliptin
Anticoagulants/Antiplatelets/ Antianemic	apixaban, cilostazol, clopidogrel, dabigatran, epoetin alfa, ferrous sulfate, heparins, polysaccharide iron, rivaroxaban, ticagrelor, warfarin
Sympatholytic agents	alfuzosin, tamsulosin
Sympathomimetic agents	albuterol, albuterol/ipratropium, atropine/chlorpheniramine/hyoscyamine/ pseudoephedrine/scopolamine, budesonide/formoterol, carbinoxamine/methscopolamine/pseudoephedrine, chlorpheniramine/acetaminophen/phenylephrine, cinnamedrine, codeine/guaifenesin/ pseudoephedrine, dexbrompheniramine/pseudoephedrine, epinephrine, fluticasone/vilanterol fluticasone/umeclidinium/vilanterol, formoterol/mometasone, fluticasone/salmeterol, olodaterol/tiotropium, pseudoephedrine, pseudoephedrine/acetaminophen, salmeterol umeclidinium/vilanterol
Anticonvulsants	carbamazepine, clonazepam, gabapentin, lamotrigine, levetiracetam, pregabalin, primidone<br>topiramate
Notes: All brand names are converted to generic names	

*This table only includes PDI medications endorsed by the study participants. We did not include all medicines that could potentially affect driving performance.

© 2023 Carr DB et al. *JAMA Network Open.*

eTable 3. Incidence rates and adjusted hazard ratios for risk of marginal/fail rating on road test (i.e. event) by PDI drug categories (N=198)

Drug[a]	No. in each group	Time at risk (years)	No. of marginal /fail rating[b]	No. of marginal/fail rating per 100 person-year	HR (95% CI)[c.d]	p-value
**Anticoagulants/Antiplatelets**						
No	172	725.3	62	8.5	0.68	0.330
Yes	26	135.1	8	5.9	(0.32, 1.47)	
**Anticonvulsants**						
No	172	751.0	58	7.7	1.27	0.478
Yes	26	109.3	12	11.0	(0.65, 2.47)	
**Antidiabetics**						
No	177	780.0	62	7.9	1.21	0.632
Yes	21	80.4	8	9.9	(0.56, 2.62)	
**Lipid-lowering agents**						
No	86	369.8	38	10.3	0.67	0.123
Yes	112	490.6	32	6.5	(0.41, 1.11)	
**Sympathomimetic agents**						
No	162	710.6	57	8.0	0.85	0.604
Yes	36	149.8	13	8.7	(0.45, 1.60)	
**Sympatholytic agents**						
No	174	755.5	58	7.7	1.86	0.090
Yes	24	105.0	12	11.4	(0.91, 3.83)	

- [a]For all drugs, "No" response option served as the reference group for Cox proportional hazards regression analyses
- [b]No. of marginal/fail rating refers to the number of participants who received marginal/fail rating on a road test.
- [c]For Cox regression, marginal/fail rating on road test (No=0) served as the reference group
- [d]Hazard ratios were adjusted for sex, age, education, ADI, CCI, PACC, and Visual acuity far score
- SSRIs/SNRIs = Selective serotonin reuptake inhibitors/Serotonin and norepinephrine reuptake inhibitors
- NSAIDs = Non-steroidal anti-inflammatory drugs

© 2023 Carr DB et al. *JAMA Network Open.*

eTable 4. Incidence rates and adjusted hazard ratios for risk of marginal/fail rating on road test (i.e. event) by PDI drug categories (N=198)

Drug[a]	No. in each group	Time at risk (years)	No. of marginal /fail rating[b]	No. of marginal/fail rating per 100 person-year	HR (95% CI)[c,d]	p-value
**Medications included in primary analysis**						
Any Antidepressant						
No	151	695.7	44	6.3	2.88	<0.00
Yes	47	164.7	26	15.8	(1.72, 4.81)	1
SSRI/SNRI						
No	165	746.6	50	6.7	2.74	<0.00
Yes	33	113.8	20	17.6	(1.58, 4.75)	1
Sedative and hypnotic agents						
No	177	788.6	58	7.4	2.47	0.007
Yes	21	71.8	12	16.7	(1.28, 4.80)	
NSAIDs/Acetaminophen						
No	51	205.4	9	4.4	2.31	0.021
Yes	147	655.0	61	9.3	(1.13, 4.70)	
Anticholinergic/Antihistamines						
No	114	469.3	42	8.9	0.81	0.417
Yes	84	391.1	28	7.2	(0.50, 1.34)	
**Medications included in secondary analysis**						
Anticoagulants/Antiplatelets						
No	172	725.3	62	8.5	0.60	0.197
Yes	26	135.1	8	5.9	(0.28, 1.30)	
Anticonvulsants						
No	172	751.0	58	7.7	1.21	0.569
Yes	26	109.3	12	11.0	(0.62, 2.37)	
Antidiabetics						
No	177	780.0	62	7.9	1.26	0.553
Yes	21	80.4	8	9.9	(0.587, 2.71)	
Lipid-lowering agents						
No	86	369.8	38	10.3	0.64	0.078
Yes	112	490.6	32	6.5	(0.39, 1.05)	
Sympathomimetic agents						
No	162	710.6	57	8.0	0.80	0.502
Yes	36	149.8	13	8.7	(0.42, 1.52)	
Sympatholytic agents						
No	174	755.5	58	7.7	1.97	0.068
Yes	24	105.0	12	11.4	(0.95, 4.08)	

© 2023 Carr DB et al. *JAMA Network Open.*

**Total**		198	860.4	70	8.1	-	-

- [a]For all drugs, "No" response option served as the reference group for Cox proportional hazards regression analyses
- [b]No. of marginal/fail rating refers to the number of participants who received marginal/fail rating on a road test.
- [c]For Cox regression, marginal/fail rating on road test (No=0) served as the reference group
- [d]Hazard ratios were adjusted for sex, age, education, ADI, CCI, PACC, and Visual acuity far score
- SSRIs/SNRIs = Selective serotonin reuptake inhibitors/Serotonin and norepinephrine reuptake inhibitors
- NSAIDs = Non-steroidal anti-inflammatory drugs

© 2023 Carr DB et al. *JAMA Network Open.*

eTable 5. Adjusted hazard ratios for marginal/fail rating on a road test estimated with the Fine–Gray subdistribution hazard analysis (N=198)

**Drug**[a]	**SHR (95% CI)**[b,c]	**p-value**
**Medications included in primary analysis**		
Any Antidepressant	2.91 (1.75, 4.86)	<0.001
SSRI/SNRI	2.75 (1.61, 4.70)	<0.001
Sedative and hypnotic agents	2.72 (1.32, 5.62)	0.007
NSAIDs/Acetaminophen	2.63 (1.30, 5.35)	0.007
Anticholinergic/Antihistamines	0.88 (0.53, 1.46)	0.620
**Medications included in secondary analysis**		
Anticoagulants/Antiplatelets	0.67 (0.33, 1.35)	0.264
Anticonvulsants	1.22 (0.59, 2.50)	0.593
Antidiabetics	1.08(0.48, 2.40)	0.860
Lipid-lowering agents	0.66 (0.40, 1.10)	0.108
Sympathomimetic agents	0.89 (0.45, 1.74)	0.731
Sympatholytic agents	1.88 (0.94, 3.76)	0.074

- [a]For all drugs, "No" response option served as the reference group for Competing-risks regression analyses
- [b]Subdistribution hazard ratios (SHR) were adjusted for sex, age, education, ADI, CCI, PACC, and Visual acuity far score
- [c]For competing-risks regression, marginal/fail rating on road test (No=0) served as the reference group
- SSRIs/SNRIs = Selective serotonin reuptake inhibitors/Serotonin and norepinephrine reuptake inhibitors
- NSAIDs = Non-steroidal anti-inflammatory drugs

© 2023 Carr DB et al. *JAMA Network Open.*
