# Supplementary material for: Medication and Road Test Performance Among Cognitively Healthy Older Adults
Source: JAMA Netw Open. 2023 Sep 29;6(9):e2335651. doi: 10.1001/jamanetworkopen.2023.35651 (PMC10543136; doi:10.1001/jamanetworkopen.2023.35651)
Supplement: Supplement 2. — Data Sharing Statement [file jamanetwopen-e2335651-s002.pdf]

Data Sharing Statement

Carr. Medication and Road Test Performance Among Cognitively Healthy Older Adults. *JAMA Netw Open*. Published September 29, 2023. doi:10.1001/jamanetworkopen.2023.35651

Data
Data available: No

Additional Information
Explanation for why data not available: Data will be available upon request.
